# Supplementary material for: vamos: variable-number tandem repeats annotation using efficient motif sets
Source: Genome Biol. 2023 Jul 27;24:175. doi: 10.1186/s13059-023-03010-y (PMC10373352; doi:10.1186/s13059-023-03010-y)
Supplement: Supplementary file 1 — Additional file 1: Theorem S1 proves that if efficient motif set exists, the replacement annotation is a good approximation of the original raw sequence, with the divergence bounded by Δ. [file 13059_2023_3010_MOESM1_ESM.pdf]

Theorem S1. *Let Σ^* denote the complete set of sequences that are concatenations of motifs from Σ. If an efficient motif set $\widetilde{\Sigma} \subseteq \Sigma$ exists, which minimizes the sum of $\lambda \times ||\widetilde{\Sigma}||$ and total motifs replacement cost $\sum_{i=1}^{p} o_i * \sum_{j=1}^{p} x_{ij} * \delta_{ij}$ and satisfies the three requirements below, then there exists $\widetilde{v_j} \in \widetilde{\Sigma}^*$ such that $\sum_j div(v_j, \widetilde{v_j}) \leq \sum_{i=1}^{p} o_i * \sum_{j=1}^{p} x_{ij} * \delta_{ij} < \Delta, v_j \in V$.*

- *All occurrence of $m_i \in \Sigma$ is replaced by one and only one $m_j \in \widetilde{\Sigma}$ (possibly itself).*
- *Motif m_i cannot be replaced by motif m_j if $o_i \geq o_j$.*
- *The total motifs replacement cost $\sum_{i=1}^{p} o_i * \sum_{j=1}^{p} x_{ij} * \delta_{ij} < \Delta$.*

Proof. Assume an efficient motif set $\widetilde{\Sigma} \subseteq \Sigma$ exists. Each VNTR sequence v_j can be represented as a sequence of original motifs $m_j^1 \circ \cdots \circ m_j^l$, each $m_j^i \subseteq \Sigma$. By substituting each m_j^i with its counterpart efficient motif $\widetilde{m_j^i}$, we get $\widetilde{v_j} = \widetilde{m_j^1} \circ \cdots \circ \widetilde{m_j^l}$. For each v_j and $\widetilde{v_j}$, it is clear that $div(v_j, \widetilde{v_j}) \leq \sum_i div(m_j^i, \widetilde{m_j^i})$, otherwise the combination of edit operations from individual motif alignments would infer a more parsimonious edit distance for v_j and $\widetilde{v_j}$. Therefore, $\sum_{j=1} div(v_j, \widetilde{v_j}) \leq \sum_i div(m_j^i, \widetilde{m_j^i}) = \sum_{i=1}^{p} o_i * \sum_{j=1}^{p} x_{ij} * \delta_{ij} < \Delta$. □
