# Supplementary material for: vamos: variable-number tandem repeats annotation using efficient motif sets
Source: Genome Biol. 2023 Jul 27;24:175. doi: 10.1186/s13059-023-03010-y (PMC10373352; doi:10.1186/s13059-023-03010-y)
Supplement: Supplementary file 2 — Additional file 2: Theorem S2 proves that the efficient motif set selection problem is a NP-hard problem when the cost function div is a general function not limited to edit distance. [file 13059_2023_3010_MOESM2_ESM.pdf]

Theorem S2. *The efficient motif set selection problem is NP-hard.*

Proof. To prove the efficient motif set selection problem is NP-hard, we can prove a special case of the problem is NP-hard. When λ is set to ∞, the minimization objective turns into simply minimizing the size of the efficient motif set. When all motif counts o_i are the same, the second requirement in Theorem S1 (Additional file 1) will always be satisfied.

The decision version of simplified efficient motif set selection problem can be formulated in the following way: given a k, can we find a subset $\widetilde{\Sigma} \subseteq \Sigma$ with $||\widetilde{\Sigma}|| \leq k$, such that the total replacing cost $\sum_{i=1}^{p} o_i * x_{ij} * \delta_{ij} < \Delta$. The decision version of the set cover problem can be formulated as the following: given a k, a set of elements $\{e_1, e_2, ..., e_p\}$ and a collection of p subsets $\{S_1, S_2, ..., S_p\}$ of elements, whose union equals the universe, can we find a sub-collection of p sets whose union equals the universe and the size of the sub-collection is less than or equal to k.

To prove the optimization version of the problem is NP-hard, we can prove the decision version of simplified efficient motif selection problem is NP-complete. To prove the NP-completeness of the decision version, first, we show the problem belongs to NP.

Suppose we have a solution of variables $\{x_{ij}\}_{i,j=1,...,p}$, we can check if $||\widetilde{\Sigma}|| \leq k$ and the total cost $\sum_{i=1}^{p} o_i * \sum_{j=1}^{p} \delta_{ij} x_{ij} < \Delta$ in polynomial time. Second, we prove the problem is NP-complete by reducing the decision version of the special set cover problem with equal size of elements and sets to it.

Let a set cover problem instance be a set of p elements $\{e_1, e_2, ..., e_p\}$ and a collection of p sets of elements $\{S_1, S_2, ..., S_p\}$ whose union equals the universe of elements. For any set cover problem instance, we can create an instance of efficient motifs selection problem: define a set of p motifs as $\{m_1, m_2, ..., m_p\}$ and associated counts $o_i = 1$ for $\forall i = 1, ..., p$. Set $\Delta = 0.5$ and $\delta_{ij} = \mathbb{1}(e_i \notin S_j)$.

Suppose $\{x_{ij}\}_{i,j=1,...,p}$ is a solution of the constructed instance of the efficient motifs selection problem. We can prove a sub-collection of sets which is defined as $\{S_t\}_{t \in Sub}$, $Sub = \{j \in \{1, ..., p\} | \mathbb{1}(\sum_{i=1}^{p} x_{ij} \geq 1)\}$ is a valid solution of the set cover instance.

- Since $||\widetilde{\Sigma}|| = \sum_{j=1}^{p} \mathbb{1}(\sum_{i=1}^{p} x_{ij} \geq 1) \leq k$, thus $||Sub|| \leq k$.
- Since each motif m_i must have a replacement, there exists some j such that $x_{ij} = 1$. Since $\sum_{i=1}^{p} \sum_{j=1}^{p} \delta_{ij} x_{ij} < \Delta = 0.5$ and $\delta_{ij} = \mathbb{1}(e_i \notin S_j)$, then $x_{ij} = 1 \implies \delta_{ij} = 0 \implies e_i \in S_j$. Additionally by the definition of Sub, such $S_j \in \{S_t\}_{t \in Sub}$. Therefore, for each e_i, there exists $S_j \in \{S_t\}_{t \in Sub}$ such that $e_i \in S_j$.

Next, suppose a sub-collection $\{S_t\}_{t \in Sub}, Sub \subseteq \{1, ..., p\}$ is a solution of the set cover problem instance. We define $\{x_{ij}\}_{i,j=1,...,p}$ (variables indicating if motif m_i is replaced by m_j) as follows:

- If $j \notin Sub$, $x_{ij} = 0$.
- If $j \in Sub$ and $e_i \notin S_j$, $x_{ij} = 0$.

- If $j \in Sub$, $e_i \in S_j$, and S_j is the only set in $\{S_t\}_{t\in Sub}$ that covers e_i, then $x_{ij} = 1$.
- If $j \in Sub$, $e_i \in S_j$, and there are more than one set in $\{S_t\}_{t\in Sub}$ that covers e_i, suppose $S_{j_{min}}$ has the minimum index among these sets, set $x_{ij_{min}} = 1$; for any other $S_{j'}$, set $x_{ij'} = 0$.

We next prove the defined $\{x_{ij}\}_{i,j=1,\ldots,p}$ is a valid solution of the constructed instance of the efficient motif selection problem.

- $||\widetilde{\Sigma}|| = \sum_{j=1}^{p} \mathbb{1}(\sum_{i=1}^{p} x_{ij} \geq 1) \leq ||Sub|| \leq k$.
- For each e_i, there must exist some j such that $e_i \in S_j$. So there must exist some j' (not necessarily j) such that $x_{ij'} = 1$. And according to the definition, there is only one j' such that $x_{ij'} = 1$.
- For every i, j, since $x_{ij} = 1 \Longrightarrow e_i \in S_j \Longrightarrow \delta_{ij} = 0$, $\sum_{i=1}^{p}\sum_{j=1}^{p} \delta_{ij} x_{ij} = 0 < \Delta = 0.5$.

To sum up, we prove the decision version of simplified efficient motif selection problem is NP-complete, thus the optimization version of the problem is NP-hard. Therefore, efficient motif selection problem is NP-hard. □
